# Sinomenine Hydrochloride Can Ameliorate Benign Prostatic Hyperplasia by Lowering the 5α-Reductase 2 Level and Regulating the Balance between the Proliferation and Apoptosis of Cells

**DOI:** 10.3390/molecules28020803

**Published:** 2023-01-13

**Authors:** Mao-Si Fan, Yue-Fei Xia, Rui-Han Ye, Ze-Rui Sun, Ming-Yue Wang, Meng-Fei An, Shao-Shi Zhang, Li-Juan Zhang, Yun-Li Zhao, Ze-Min Xiang, Jun Sheng

**Affiliations:** 1 Key Laboratory of Pu-erh Tea Science, Ministry of Education, College of Science, Yunnan Agricultural University, Kunming 650224, China; 2 College of Food Science and Technology, Yunnan Agricultural University, Kunming 650224, China; 3 Chinese Materia Medica, Yunnan University of Chinese Medicine, Kunming 650500, China; 4 School of Basic Medicine, Yunnan University of Chinese Medicine Chinese, Kunming 650500, China; 5 Key Laboratory of Medicinal Chemistry for Natural Resource, Ministry of Education and Yunnan Province, Yunnan Characteristic Plant Extraction Laboratory, Yunnan Provincial Center for Research & Development of Natural Products, School of Pharmacy, School of Chemical Science and Technology, Yunnan University, Kunming 650500, China; 6 State Key Laboratory for Conservation and Utilization of Bio-Resources in Yunnan, Kunming 650051, China

**Keywords:** benign prostatic hyperplasia, sinomenine hydrochloride, 5α-reductase 2, apoptosis, PCNA

## Abstract

Benign prostatic hyperplasia (BPH) is a chronic disease that affects the quality of life of older males. Sinomenine hydrochloride (SIN) is the major bioactive alkaloid isolated from the roots of the traditional Chinese medicinal plant *Sinomenium acutum Rehderett Wilson*. We wondered if the SIN administration exerted a regulatory effect on BPH and its potential mechanism of action. Mice with testosterone propionate-induced BPH subjected to bilateral orchiectomy were employed for in vivo experiments. A human BPH cell line (BPH-1) was employed for in vitro experiments. SIN administration inhibited the proliferation of BPH-1 cells (*p* < 0.05) by regulating the expression of androgen-related proteins (steroid 5-alpha reductase 2 (SRD5A2), androgen receptors, prostate-specific antigen), apoptosis-related proteins (B-cell lymphoma 2 (Bcl-2), Bcl-2-associated X protein (Bax)) and proliferation-related proteins (proliferating cell nuclear antigen (PCNA), mammalian target of rapamycin, inducible nitric oxide synthase) in vitro. SIN administration decreased the prostate-gland weight coefficient (*p* < 0.05) and improved the histological status of mice suffering from BPH. The regulatory effects of SIN administration on SRD5A2, an apoptosis-related protein (Bcl-2), and proliferation-related proteins (PCNA, matrix metalloproteinase-2) were consistent with in vitro data. SIN exerted a therapeutic effect against BPH probably related to lowering the SRD5A2 level and regulating the balance between the proliferation and apoptosis of cells. Our results provide an important theoretical basis for the development of plant medicines for BPH therapy.

## 1. Introduction

Benign prostatic hyperplasia (BPH) is one of the most common diseases in older men [1]. BPH is detected in >50% of men over the age of 50 years, whereas ≥90% of men over the age of 80 years have BPH symptoms [2].

Abnormal proliferation of prostatic epithelial cells and stromal cells is the cause of hypertrophy of the prostate gland (PG). This action increases the tension in the smooth muscle of the PG, promotes the narrowing of the urethral cavity, increases resistance in the bladder outlet, and leads to lower urinary tract symptoms (LUTS) [3]. LUTS include frequent urination, urgency, nocturnal enuresis, and double micturition, which seriously affect the quality of life of patients [4]. 

Drugs such as inhibitors of 5α-reductase [5] and blockers of α-adrenergic receptors [6] have been shown to be efficacious in BPH treatment. However, these therapies have some side-effects including erectile dysfunction [7], bladder-neck relaxation [8], abnormal ejaculation [9], dizziness, and insomnia. Therefore, the development of alternative agents that can exert long-term therapeutic effects and reduce undesired problems in clinical use is needed. Increasing research is being conducted to develop new drugs from natural sources with potential therapeutic effects and fewer side-effects.

**Figure 1:**
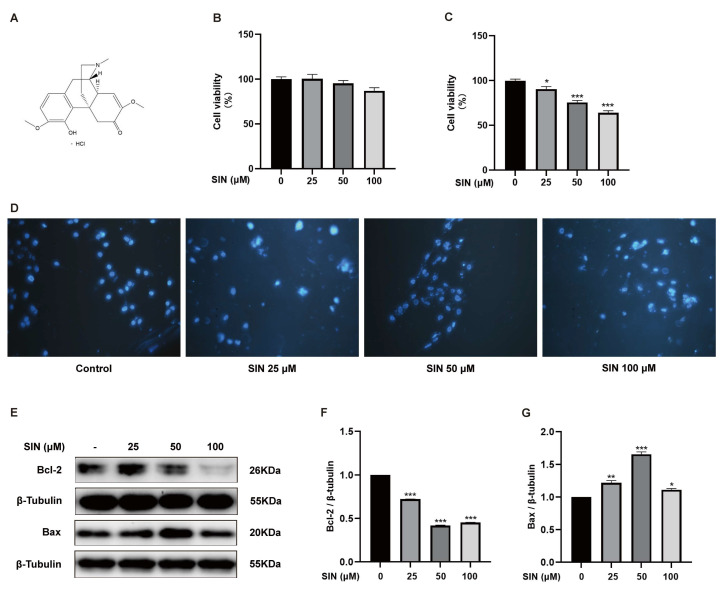
Effect of SIN on BPH-1 cell apoptosis. (**A**) Chemical structure of SIN. (**B**) Effects of SIN on RWPE-1 cell viability. (**C**) Effects of SIN on BPH-1 cell viability. (**D**) Representative images of DAPI-stained cells were observed under a fluorescence microscope. (**E**) Effects of SIN on the expression of apoptosis-related proteins. Protein quantification for (**F**) Bcl-2, (**G**) Bax versus *β*-tubulin. Data are the mean ± SEM. Statistics: * *p* < 0.05, ** *p* < 0.01, *** *p* < 0.001 versus the control group. SIN, sinomenine hydrochloride; Bcl-2, B-cell lymphoma-2; Bax, BCL2-associated X protein.

Herbal extracts have modest efficacy and are considered complementary or alternative treatments for BPH [10]. Several promising herbal extracts against BPH have been reported, including epimedium [11], Red Maca [12], and Asteris Radix et Rhizoma [13]. *Serenoa repens* (known commonly as “saw palmetto”) is a well-studied herbal extract for BPH treatment [14]. Sinomenine hydrochloride (SIN) is the hydrochloride form of sinomenine. SIN is the major bioactive alkaloid isolated from the root of the traditional Chinese medicinal plant *Sinomenium acutum Rehderett Wilson*. SIN (Figure 1A) is a drug approved by theNational Medical Products Administration and used widely for many years in China for the treatment of rheumatoid arthritis [15]. Reports on the therapeutic effect of SIN on BPH are lacking.

Androgens promote the proliferation of epithelial cells or stromal cells within the PG in autocrine or paracrine manners. This action leads to an imbalance of the proliferation and apoptosis of PG cells, which is considered to be an important cause of BPH [16]. In the PG, testosterone is converted to dihydrotestosterone (DHT) by the enzyme steroid 5-alpha reductase 2 (SRD5A2), which is a potent androgen because of its high binding ability to androgen receptors (ARs) [17]. Testosterone and DHT bind to ARs, resulting in increased transcription of androgen-dependent genes and, ultimately, the stimulation of protein synthesis [18]. In addition, DHT enhances the prostate-specific antigen (PSA) level by binding to ARs. The PSA level is increased during BPH and in prostate cancer. Therefore, the PSA level is used widely to assist in BPH diagnosis [19]. 

The cell cycle in prostatic stromal and epithelial cells leads to their division and replication. Proliferating cell nuclear antigen (PCNA) is a histological marker of the G1/S phase of the cell cycle, which can reflect the proliferation of PG cells during BPH [20]. Apoptosis of prostatic epithelial cells occurs more frequently in health than during BPH. Apoptosis is a form of programmed cell death triggered by the regulation of the ratio of anti-apoptotic factors to pro-apoptotic factors [21]. The anti-apoptotic protein B-cell lymphoma-2 (Bcl-2) inhibits apoptosis and the pro-apoptotic protein Bcl-2-associated X protein (Bax) promotes apoptosis [22].

We examined the modulatory effects and mechanism of action of SIN on BPH in vitro and in vivo using a BPH cell line and mice with testosterone propionate (TP)-induced BPH, respectively.

## 2. Results

### 2.1. Effect of SIN Treatment on the Apoptosis of BPH-1 Cells

To assess the effect of SIN therapy on BPH, we first used normal PG epithelial (RWPE-1) cells and BPH (BPH-1) cells to select the SIN concentration for subsequent cell experiments. Treatment of RWPE-1 cells and BPH-1 cells with SIN (25, 50, 100 μM) for 48 h did not lead to a significant inhibitory effect on RWPE-1 cells (*p* > 0.05) (Figure 1B) but inhibited BPH-1 cells significantly in a dose-dependent manner (*p* < 0.05) (Figure 1C). To further elucidate the growth-inhibitory effect of SIN on BPH-1 cells, we investigated the apoptosis of BPH-1 cells using DAPI staining of nuclei. BPH-1 cells that did not receive SIN treatment had intact nuclei and no wrinkles. BPH-1 cells treated with SIN did not show the obvious signs of apoptosis (e.g., formation of apoptotic bodies and nuclear rupture) (Figure 1D). Subsequently, we measured the protein expression of apoptosis-regulatory markers in BPH-1 cells via western blotting. In BPH-1 cells, SIN therapy inhibited the protein expression of Bcl-2 significantly (*p* < 0.001) (Figure 1F) and enhanced the protein expression of Bax in BPH-1 cells significantly (*p* < 0.05) (Figure 1G). Our data supported the theory that SIN inhibits the proliferation of BPH-1 cells through the apoptotic pathway.

### 2.2. Effect of SIN Treatment on the AR Signaling Pathway in BPH-1 Cells

**Figure 2:**
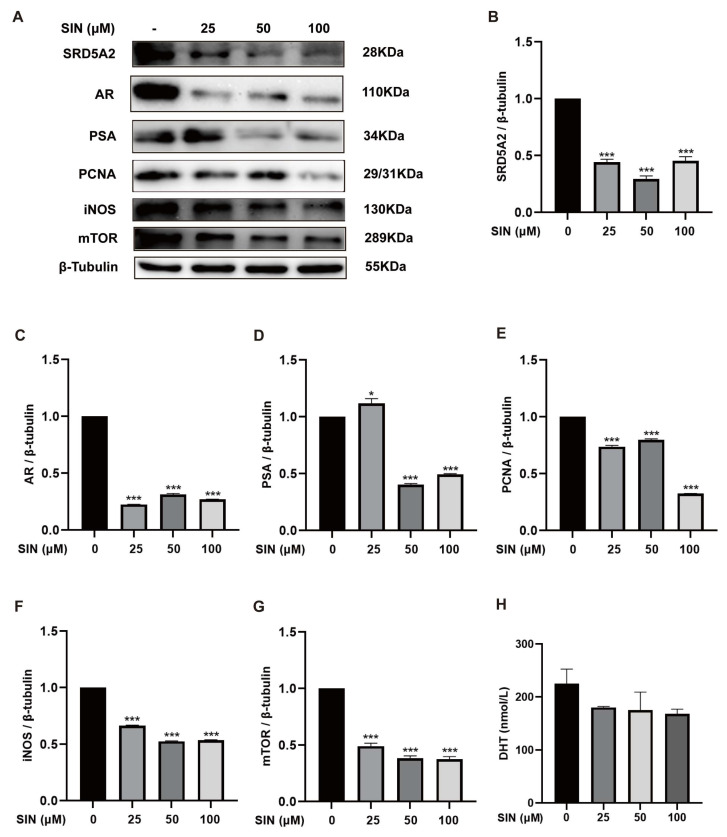
Effect of SIN administration on the AR signaling pathway in BPH-1 cells. (**A**) Effects of SIN treatment on expression of AR signaling pathway-related proteins. Protein quantification for (**B**) SRD5A2, (**C**) AR, (**D**) PSA, (**E**) PCNA, (**F**) iNOS, and (**G**) mTOR versus *β*-tubulin. (**H**) DHT level in cell supernatants. Data are the mean ± SEM. * *p* < 0.05, *** *p* < 0.001 versus the control group. SIN, sinomenine hydrochloride; SRD5A2, steroid 5-alpha reductase 2; AR, androgen receptor; PSA, prostate-specific antigen; PCNA, proliferating cell nuclear antigen; iNOS, inducible nitric oxide synthase; mTOR, mammalian target of rapamycin; DHT, dihydrotestosterone.

To investigate the role of SIN in the androgen signaling of BPH, protein expression of SRD5A2, AR, and PSA in BPH-1 cells was measured via western blotting and in cell supernatants by ELISAs. Overexpression of SRD5A2 (*p* < 0.001) (Figure 2B), AR (*p* < 0.001) (Figure 2C), and PSA (*p* < 0.001) (Figure 2D) in BPH-1 cells was downregulated by SIN treatment (Figure 2A). SIN therapy induced a reduction in the DHT concentration measured in the supernatants of BPH-1 cells, though the difference was not significant (*p* > 0.05) (Figure 2H). High protein expression of PCNA (*p* < 0.001) (Figure 2E), iNOS (*p* < 0.001) (Figure 2F), and mTOR (*p* < 0.001) (Figure 2G) was inhibited significantly by SIN treatment.

### 2.3. Effects of SIN Therapy on Prostatic Enlargement in Mice with TP-Induced BPH

**Figure 3:**
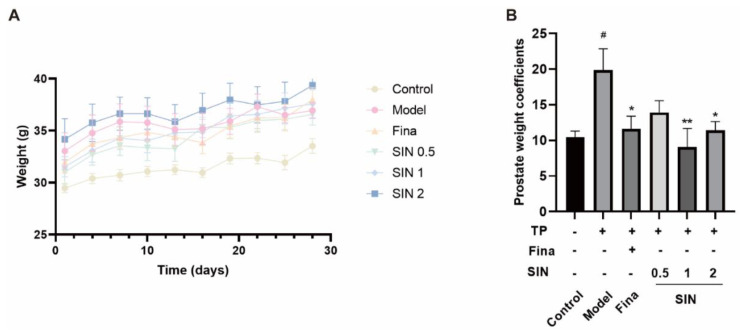
Effect of SIN administration on prostatic enlargement in mice with testosterone propionate (TP)-induced BPH. (**A**) Plot of body weight change in mice. The bodyweight of mice was recorded every 3 d. (**B**) Prostate weight coefficient in mice. Prostate-gland weight coefficient = wet weight of prostate gland (mg)/bodyweight of mice (g) × 10. Data are the mean ± SEM. ^#^ *p* < 0.05 versus the control group; * *p* < 0.05, ** *p* < 0.01 versus the model group. Fina, finasteride; SIN, sinomenine hydrochloride.

To confirm the anti-BPH effect of SIN treatment in vivo, a mouse model of BPH was established by administering finasteride or SIN (0.5, 1, 2 mg/kg) concomitant with TP injection for 4 weeks. Bodyweight was measured every 3 days to determine the effects of TP and finasteride/SIN treatments (Figure 3A). Mice organs were weighed to ascertain the effects of SIN treatment on TP-induced BPH in mice. The PG weight coefficient (i.e., wet weight of PG (mg)/bodyweight of mice (g) × 10) in mice of the model group was significantly higher than that of the control group (19.87 vs. 10.43 mg/10 g, *p* < 0.05) (Figure 3B). The finasteride group had a significantly lower PG weight coefficient compared with that of the model group (11.63 mg/10 g, *p* < 0.05) (Figure 3B). Compared with the model group, the PG weight coefficient of SIN groups (0.5, 1, 2 mg/kg) was reduced to 13.92, 9.05, and 11.41 mg/10 g, respectively (Figure 3B).

### 2.4. Effects of SIN Treatment on Protein Expression in the PG Tissue of Mice with TP-Induced BPH

**Figure 4:**
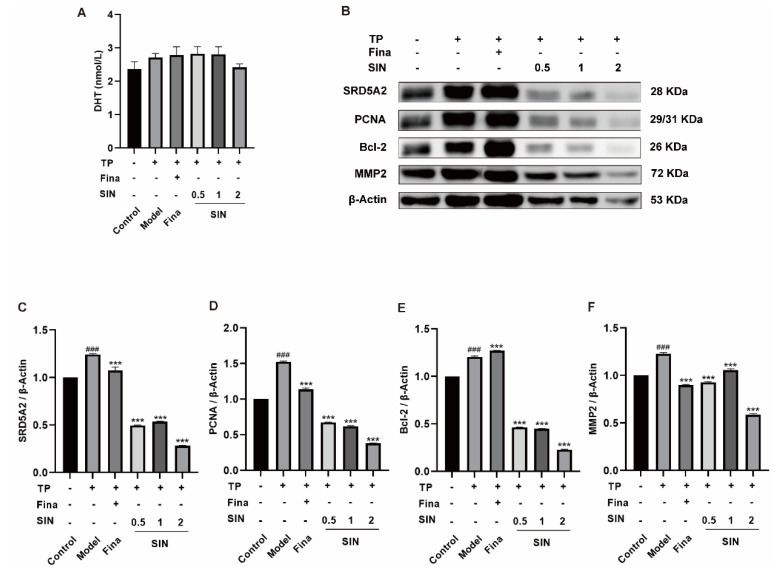
Effect of SIN administration on the expression of related proteins in the PG tissue of mice with TP-induced BPH. (**A**) The level of DHT in serum. (**B**) Effect of SIN administration on the expression of related proteins in the PG tissue of mice with TP-induced BPH. Protein quantification for (**C**) SRD5A2, (**D**) PCNA, (**E**) Bcl-2, and (**F**) MMP2 versus *β*-tubulin. Data the means ± SEM. ^###^ *p* < 0.001 versus the control group; *** *p* < 0.001 versus the model group. Fina, finasteride; SIN, sinomenine hydrochloride; SRD5A2, steroid 5 alpha-reductase 2; PCNA, proliferating cell nuclear antigen; Bcl-2, B-cell lymphoma-2; MMP2, matrix metallopeptidase 2.

As shown in Figure 4A, the DHT in the serum of the model group was higher than that of the control group (2.71 vs. 2.37 nmol/L, *p* > 0.05). Interestingly, the high dose of SIN (2 mg/kg) decreased the content of DHT (*p* > 0.05). The overexpression of SRD5A2 (*p* < 0.001) (Figure 4C), PCNA (*p* < 0.001) (Figure 4D), Bcl-2 (*p* < 0.001) (Figure 4E), and MMP2 (*p* < 0.001) (Figure 4F) in the PG tissue of mice with TP-induced BPH was downregulated by treatment with finasteride or SIN. Moreover, the effects of SIN on protein expression of PCNA and Bcl-2 were dose-dependent.

### 2.5. Effects of SIN Therapy on Histological Alterations of PG Tissue in Mice with TP-Induced BPH

**Figure 5:**
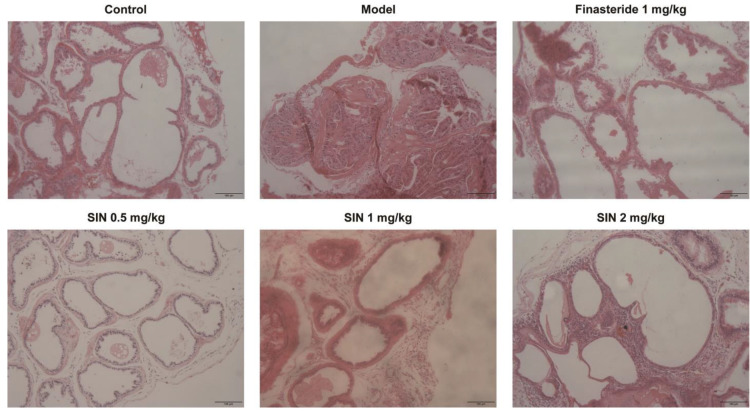
Effect of SIN administration on histological variations of prostate-gland tissue in mice with TP-induced BPH. Representative micrographs of prostate-gland tissue stained with H&E (×200 magnification), scale bar = 100 μm. SIN, sinomenine hydrochloride.

Histological alterations in the PG tissue of mice with TP-induced BPH were examined via H&E staining. Compared with the control group, the number of layers of glandular epithelial cells, volume, folds in the acinar structure, area of the glandular lumen, epithelial-cell proliferation, and infiltration of some inflammatory cells were increased significantly in the model group. Cells were restored to a morphology similar to that of the control group following the administration of finasteride or SIN (0.5, 1, 2 mg/kg) (Figure 5).

## 3. Discussion

BPH and related LUTS are common urinary-system problems in older men, which place a huge burden on their health and quality of life. SIN is used widely in the clinical treatment of rheumatoid diseases because of its anti-inflammatory and anti-immune effects. We designed experiments to explore the regulatory effects of SIN therapy on BPH based on cellular and animal models. BPH-1 cells were used as the in vitro model. Mice with TP-induced BPH subjected to bilateral orchiectomy were used as the in vivo model. Bilateral orchiectomy in mice prevented interference by endogenous androgens, and a model of androgen-dependent BPH was generated by subcutaneous injection of TP. In TP-treated mice with bilateral testes excision, the PG weight and glandular epithelial layer were increased significantly, along with folds in the acinar structure, epithelial-cell proliferation, and infiltration by certain inflammatory cells (Figure 5), data which are consistent with the results of work by Karunasagara [23] and colleagues. In vitro, SIN therapy inhibited the proliferation of BPH-1 cells, but not RWPE-1 cells, which suggested the safety of SIN treatment. The improvement effect of SIN therapy on BPH in vitro was consistent with in vivo results.

Changes in androgen levels during aging are thought to be an important cause of BPH [24]. Approximately 90% of the PG androgen DHT is converted from testosterone by the catalysis elicited by SRD5A2 [25]. Once DHT binds to an AR, its affinity is about 2–5-times higher than that of testosterone, which induces PSA to induce BPH [26]. The androgen signaling pathway plays an important part in BPH development. SIN therapy downregulated protein expression of SRD5A2 (Figure 2B), AR (Figure 2C), and PSA (Figure 2D) significantly in BPH-1 cells, and SIN treatment decreased the DHT concentration in the supernatants of BPH-1 cells (Figure 2H), which suggested that SIN therapy inhibited the proliferation of BPH-1 cells through the androgen signaling pathway. In vivo, SIN treatment reduced protein expression of SRD5A2 significantly in the PG tissue of mice with TP-induced BPH (Figure 4B), and SIN tended to decrease DHT in serum (Figure 4A), a finding that was consistent with data in the in vivo study. A complex regulatory relationship between androgens and growth factors exists, which changes in response to hormonal changes. mTOR is an atypical serine/threonine kinase. It promotes cell growth by phosphorylating substrates to enhance anabolism or limit catabolism [27]. SIN treatment reduced the protein expression of mTOR in BPH-1 cells (Figure 2G).

The most prominent features of BPH have increased cell proliferation and decreased apoptosis. Therefore, enhancing apoptosis in BPH is a therapeutic strategy. Intrinsic apoptosis is regulated by the anti-apoptotic protein Bcl-2, a protein located in the outer mitochondrial membrane that inhibits the release of the pro-apoptotic factor cytochrome C [28]. The Bax gene is a protein of the Bcl-2 family that activates apoptotic signals. In health, PG tissue has relatively low Bcl-2 expression, which is associated with a low level of apoptosis. However, in BPH, upregulation of expression of the anti-apoptotic protein Bcl-2 and downregulation of expression of the pro-apoptotic protein Bax cause an imbalance between apoptosis and proliferation, which is manifested as apoptotic signaling. Expression of PCNA (marker of proliferation) is also increased in BPH [3]. SIN therapy downregulated protein expression of Bcl-2 significantly (Figure 1F), increased protein expression of Bax significantly (Figure 1G), and decreased protein expression of PCNA significantly (Figure 2E) in BPH-1 cells. These results indicate that SIN therapy inhibited the proliferation of BPH-1 cells through the apoptotic pathway. In vivo, SIN therapy downregulated protein expression of Bcl-2 (Figure 4D) and PCNA (Figure 4C) significantly in the PG tissues of mice with TP-induced BPH, indicating that SIN treatment ameliorated BPH through the apoptotic pathway, which was consistent with in vitro data. Collectively, these findings suggest that SIN treatment may improve BPH through the apoptotic pathway.

BPH is also linked to several proteins. The collagenase MMP2 breaks-down fibronectin and laminin in the basement membrane, and is involved in various physiological and pathological processes in the human body [29]. SIN administration downregulated protein expression of MMP2 in the PG tissue of TP-treated castrated mice (Figure 4E). High expression of iNOS in BPH-1 cells was reduced by SIN therapy (Figure 2F).

## 4. Materials and Methods

### 4.1. Chemicals

SIN (98.00% purity; Chemical Abstracts Service number: 6080-33-7) was purchased from Chengdu Plant Standard Pure Biotechnology (Chengdu, China). Enzyme-linked immunosorbent assay (ELISA) kits for mouse DHT were obtained from Shanghai Jining Industrial (Chengdu, China). Antibodies against SRD5A2 (catalog number: ab240005), PSA (ab76113), Bcl-2 (ab32124), Bax (ab32503), PCNA (ab29), matrix metalloproteinase-2 (MMP2; ab 235167), and the AR (ab133273) were acquired from Abcam (Cambridge, UK). 3-(4,5-dimethylthiazol-2-yl)-2,5-diphenyltetrazolium bromide (MTT) was obtained from Solarbio (Beijing, China).

### 4.2. Cell Culture

Human normal prostatic epithelial (RWPE-1) and human BPH (BPH-1) cell lines were obtained from American Type Culture Collection (Manassas, VA, USA). Cells were cultured in Dulbecco’s modified Eagle’s medium supplemented with 10% fetal bovine serum and 1% penicillin-streptomycin (penicillin (50 U/mL) and streptomycin (50 μg/mL)) in an atmosphere of 5% CO_2_ at 37 °C.

### 4.3. Cell-Viability Assay

RWPE-1 cells and BPH-1 cells were seeded in 96-well tissue-culture plates at 5 × 10^4^ cells/well. After adherence had been confirmed, they were treated with SIN (25, 50, 100 μmol/L) for 48 h, followed by 0.4% MTT (20 μL). After incubation for 4 h in the dark, dimethyl sulfoxide (200 μL) was added to dissolve the remaining MTT formazan crystals for 10 min with agitation. Cell viability was measured at 492 nm using a multimode microplate reader (Flex Station™ 3; Molecular Devices, Silicon Valley, CA, USA). 

### 4.4. Staining

BPH-1 cells were seeded in 12-well tissue-culture plates at 3 × 10^5^ cells/well, mounted under microscope cover glasses, and treated with SIN (25, 50, 100 μmol/L) for 48 h. After treatment, the medium was discarded and cells were washed thrice with phosphate-buffered saline (PBS). After fixing with 4% paraformaldehyde for 15 min at room temperature. The removed microscope cover glasses were sealed on adhesion microscope slides with 4′,6-diamidino-2-phenylindole (DAPI) solution, and images acquired via laser scanning confocal microscopy using a setup from Leica Microsystems (Wetzlar, Germany).

### 4.5. Western Blotting 

Cells or tissues were rinsed with pre-cooled PBS and homogenized with high-efficiency radioimmunoprecipitation assay (RIPA) lysis buffer containing phenylmethylsulfonyl fluoride (100:1 dilution). Samples were placed on ice for 25 min, transferred to 1.5-mL microtubes, and centrifuged at 15,000× *g* for 10 min at 4 °C. Supernatant fractions were transferred to fully labeled 1.5-mL microtubes. Equal amounts of proteins were separated via sodium dodecyl sulfate–polyacrylamide gel electrophoresis using 8% or 10% gels and transferred to solid-phase carrier polyvinylidene fluoride (PVDF) membranes. PVDF membranes were incubated overnight at 4 °C with antibodies targeting PSA, AR, SRD5A2, PCNA, inducible nitric oxide synthase (iNOS), mammalian target of rapamycin (mTOR), Bcl-2, Bax, MMP2, β-actin, or β-tubulin, and washed four times with Tris-buffered saline containing Tween 20 for 5-min each time. PVDF membranes were incubated with horseradish peroxidase-conjugated goat anti-mouse immunoglobulin (Ig)G or goat anti-rabbit IgG antibody for 1 h at room temperature. 

### 4.6. ELISA Measurements in Cell Supernatants

BPH-1 cells were seeded in 96-well tissue-culture plates at 5 × 10^4^ cells/well. Then, they were treated with SIN (25, 50, 100 μmol/L) for 48 h. Plates were centrifuged at 3000 rpm for 15 min at 4 °C. The DHT concentration in cell supernatants was determined using a ELISA kits according to manufacturer instructions.

### 4.7. Animals

Animal-care and experimental procedures complied with international guidelines and conformed to regulations set by the Administration of Affairs Concerning Experimental Animals published by the State Science and Technology Commission of China. The study protocol was approved (YUAN2019LLWYH003-8) by the Animal Care and Use Committee of Yunnan Agricultural University (Kunming, China).

Healthy male ICR mice (25–30 g) were obtained from Cavens Biogle Model Animal Research (license number SCXK 2016-0010) in Suzhou, China. Mice were housed in conventional cages in a specific pathogen-free environment. A maximum of five mice per cage were housed in a controlled environment (ambient temperature: 22–25 °C; relative humidity: 45–55%; 12 h light–dark cycle) with free access to food and water. Animals were allowed to acclimatize to their environment for 7 days before experimentation. 

### 4.8. Mouse Model of BPH

Mice were divided randomly into six groups: control, BPH (model), finasteride treatment (1 mg/kg; positive control), and three SIN (0.5, 1, 2 mg/kg bodyweight) treatment groups. Bilateral testes were removed by castration in mice of all groups (except for the control group) to eliminate the influence of endogenous testosterone. A sham operation was undertaken under identical conditions in the control group. After a recovery period of 7 days, TP (5 mg/kg, s.c.) was injected to induce BPH in all mice (except the control group). Simultaneous with TP treatment, finasteride (1 mg/kg, i.p.) or SIN (0.5, 1, 2 mg/kg, i.p.) was injected into mice every day for 4 weeks. Mice were euthanized the day after the final dose had been administered. The PG was harvested and dissected for examination via western blotting and histopathology.

### 4.9. Histology 

PG tissues were fixed in 10% formalin, embedded in paraffin, and sliced into sections of thickness 5 μm. Then, they were stained with hematoxylin and eosin (H&E) for general morphological evaluation under a light microscope (Olympus, Tokyo, Japan), as reported previously [30].

### 4.10. Statistical Analyses 

Images were analyzed using ImageJ (US National Institutes of Health, Bethesda, MD, USA). Statistical analyses were undertaken using Prism 8 (GraphPad, La Jolla, CA, USA). Data are the mean ± standard error of the mean. One-way ANOVA was applied for data analysis at a single time-point. *p* < 0.05 was considered significant.

## 5. Conclusions

SIN exerted a therapeutic effect against BPH probably related to lowering the SRD5A2 level and regulating the balance between the proliferation and apoptosis of cells.

## Data Availability

The data presented in this study are available on request from the corresponding author.
